# A protein interaction atlas for the nuclear receptors: properties and quality of a hub-based dimerisation network

**DOI:** 10.1186/1752-0509-1-34

**Published:** 2007-07-31

**Authors:** Gregory D Amoutzias, Elgar E Pichler, Nina Mian, David De Graaf, Anastasia Imsiridou, Marc Robinson-Rechavi, Erich Bornberg-Bauer, David L Robertson, Stephen G Oliver

**Affiliations:** 1 Faculty of Life Sciences, University of Manchester, Manchester, M13 9PT, UK; 2 Department of Ecology and Evolution, University of Lausanne & Swiss Institute of Bioinformatics, 1015 Lausanne, Switzerland; 3 Discovery Information, AstraZeneca R&D Boston, 35 Gatehouse Drive, Waltham, MA 02451, USA; 4 Bioinformatics & Evolutionary Genomics, Department of Plant Systems Biology, VIB/Ghent University, Technologiepark 927, B-9052 Ghent, Belgium; 5 AstraZeneca R&D, Alderley Park, UK; 6 Pfizer RTC Cambridge, Cambridge, MA, USA; 7 Higher Technological Educational Institute of Thessaloniki, 63200 Nea Moudania, Halkidiki, Greece; 8 Bioinformatics Division, Institute for Evolution and Biodiversity, School of Biological Sciences, University of Muenster, Schlossplatz 4, D48149, Muenster, Germany

## Abstract

**Background:**

The nuclear receptors are a large family of eukaryotic transcription factors that constitute major pharmacological targets. They exert their combinatorial control through homotypic heterodimerisation. Elucidation of this dimerisation network is vital in order to understand the complex dynamics and potential cross-talk involved.

**Results:**

Phylogeny, protein-protein interactions, protein-DNA interactions and gene expression data have been integrated to provide a comprehensive and up-to-date description of the topology and properties of the nuclear receptor interaction network in humans. We discriminate between DNA-binding and non-DNA-binding dimers, and provide a comprehensive interaction map, that identifies potential cross-talk between the various pathways of nuclear receptors.

**Conclusion:**

We infer that the topology of this network is hub-based, and much more connected than previously thought. The hub-based topology of the network and the wide tissue expression pattern of NRs create a highly competitive environment for the common heterodimerising partners. Furthermore, a significant number of negative feedback loops is present, with the hub protein SHP [NR0B2] playing a major role. We also compare the evolution, topology and properties of the nuclear receptor network with the hub-based dimerisation network of the bHLH transcription factors in order to identify both unique themes and ubiquitous properties in gene regulation. In terms of methodology, we conclude that such a comprehensive picture can only be assembled by semi-automated text-mining, manual curation and integration of data from various sources.

## Background

The nuclear receptors (NRs) comprise an ancient family of transcription factors (TFs) that are found in metazoa and are involved in the regulation of development, metabolism, homeostasis, reproduction, and cell death [1]. They are prominent pharmaceutical targets for diseases such as hypertension, cancer, diabetes, cardiovascular disease, cholesterol gallstone disease, and the metabolic syndrome [2-4].

NRs form a complex and highly connected dimerisation network. They bind to DNA as monomers, homodimers and heterodimers [5,3,7]. The homodimers and heterodimers can bind to DNA elements that are oriented as palindromes, direct repeats, or even everted repeats. The two dimerisation domains, that is the DNA binding domain (DBD) and ligand binding domain (LBD) work in tandem to enable DNA binding. According to this two-step hypothesis, the LBD dimerisation interface initiates the formation of the dimer in solution. The formation of the second dimer interface within the DBD restricts the receptors to binding their cognate hormone response elements on the DNA [8,9]. The ability of NRs to bind to these differently oriented repeats increases the level of complexity. The elucidation of the dimerisation network for this family is very important because the combination of different NRs in dimers increases the number of genes that they regulate (combinatorial control), creates either permissive or non-permissive dimers, combines different signalling pathways on the same promoter, and creates competition for common heterodimeric partners. As an extra level of complexity, NRs can form non-DNA-binding dimers using different interfaces. In these interactions, an NR can function as co-activator [10] or it can repress the formation of another functional DNA-binding dimer [11,12].

Our goal is to get a global understanding of the NR dimerisation network. For such a systems biology analysis, synthetic approaches are needed, where large-scale experiments like yeast two-hybrid (Y2H) analyses [13-15], microarrays [16], protein arrays [17], and text mining of the literature [18] are integrated. While none of these experimental approaches has yet been perfected, they have revealed, for the first time, some of the statistical properties of biological systems, *e.g*. the scale-free nature of protein-protein interaction, protein-DNA interaction, and metabolic networks [19]. In these scale-free networks, a small number of proteins are highly connected (i.e. they represent hubs), whereas the majority are poorly connected (i.e. they are peripheral members of the network) [20]. It has been proposed that systems with such topologies favour fast information flow (creating a so-called 'small world') and that they respond rapidly to changes, while exhibiting robustness to mutation or inhibition [21,20,23]. An effective control of many interdependencies with a minimal number of connections is a feature of biological systems that involve gene regulation [24].

NRs are an extensively researched molecular class and, consequently, the literature corpus for NRs is very large; over 70,000 articles were retrieved when querying with the generic keyword "nuclear receptor". This fact, combined with the large number of protein family members (48 genes in humans) and of synonyms (over 100) makes the elucidation of the dimerisation network a non-trivial task. Therefore, we developed a semi-automated method to scan the literature exhaustively and to try to construct as complete a picture of the NR dimerisation network as possible. In doing this, we also integrated data from previous text-mining efforts [25] and recent human Y2H data [26-28], in order to answer questions relating to the structure and functions of the NR network.

## Results

### Integration of interaction data from various sources

Previous work by the Koegl group revealed a network of 117 interactions among NRs with specific names. The data accumulated by that group combines interaction data obtained from text mining of literature abstracts for the years 1966–2001 [25] and a Y2H screen of NRs [26]. No evaluation of the biological significance of the network topology was undertaken.

Our text-mining effort, covering abstracts of papers published between January 1993 and December 2005 retrieved 127 specific protein-protein interactions. The 127 interactions were between proteins whose specific name and not the generic name (the group name) was used to describe them, *e.g. *the generic term RXR [NR2B] could refer to any of the 3 paralogues (RXR-a [NR2B1], RXR-b [NR2B2], RXR-g [NR2B3]). Since paralogues share very similar dimerising DBD and LBD domains, it is often observed that all members of one phylogenetic group will interact with all members of another group, as is the case between the RXR-PPAR [NR2B-NR1C] and RXR-RAR [NR2B-NR1B] groups. This has also been observed for the bHLH [29] and bZIP [17] families of dimerising TFs. Nevertheless, exceptions exist – such as the MINOR [NR4A3] gene from the NR4A group, which does not interact with RXR [NR2B] proteins, while its other two paralogues, NUR77 [NR4A1] and NURR1 [NR4A2], do [30]. For this reason, we selected only interactions between proteins that have a specific name.

We integrated results from i) our text-mining effort ii) data from the most comprehensive and publicly available protein-protein interaction database, HPRD [18,31], iii) the large-scale Y2H experiment in humans [27], and iv) the previous datasets from the Koegl group [26,25], to obtain the complete dataset, with a total of 179 NR protein-protein interactions. Each of the individual sources has a certain degree of overlap with the others, but it also contributes a number of unique interactions that were not covered in any other source. The contribution of each source and the number of unique interactions is shown in table 1 and in Additional file 1.

**Table 1:** Contribution of interaction datasets. Contribution of various sources (text mining, publicly available databases and Y2H experiments) towards the complete nuclear receptor interaction dataset

**Dataset**	**Unique interactions**	**Total interactions**
Amoutzias *et al *text mining	36	127
Albert *et al *text mining	11	91
HPRD	10	55
Albers *et al *Y2H	20	47
Rual *et al *Y2H	4	33
Complete nuclear receptor interaction dataset		179

### Confidence in the protein-protein interactions

**Figure 1:**
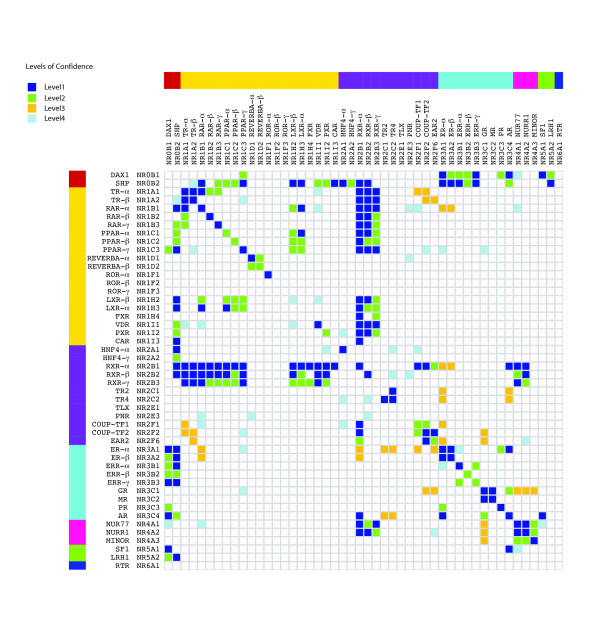
**Protein-protein interaction matrix of NRs**. Side-bar colours represent phylogenetic subfamily classifications. Colours in the matrix correspond to one of the four levels of confidence for each interaction.

Using data from literature-mining efforts (accessing scientific papers), large-scale Y2H experiments, and phylogenetics, we defined a simple measure of confidence and assigned a level of confidence to every interaction (Figure 1). The first level of confidence (L1) includes 88 interactions that have at least two different sources of evidence, (i.e. either mentioned in at least two papers, or in one paper and one of the Y2H experiments, or in two Y2H experiments). The second level of confidence (L2) includes 50 interactions between proteins that have only one source of evidence (either one paper, or one Y2H experiment), but are members of phylogenetic groups that are also linked by at least one L1 interaction. For example, the interaction between SHP [NR0B2] and RAR-gamma [NR1B3] is found only in one large-scale Y2H experiment [26] ; nevertheless, there is at least one interaction of level 1 between the NR0B and NR1B phylogenetic groups (SHP [NR0B2] – RAR-alpha [NR1B1]). The use of phylogenetic information allows us to increase the level of confidence that may be attributed to a given interaction. The third level of confidence (L3) includes 19 interactions between proteins that have only one source (one paper, or one Y2H experiment), but there also exists at least another one interaction, with only one source, between other members of these two phylogenetic groups. For example, the interaction between GR [NR3C1] and Nur77 [NR4A1] is mentioned only once in the literature, but there is at least another interaction (e.g GR [NR3C1] – Nurr1 [NR4A2]) between the NR3C and NR4A phylogenetic groups, that also has only one source of evidence. The fourth level of confidence (L4) includes 22 interactions between proteins that have only one source (one paper, or one Y2H experiment), with no other interaction between proteins of the same two phylogenetic groups. All subsequent analyses reported in this paper are performed by excluding the interactions of level 4. Evidently, the interactions attracting most confidence belong to level 1, which have been verified at least twice; whereas, levels 2 and 3 include interactions assigned moderate confidence, which have been verified by one source and are additionally supported by phylogeny. The interactions in which there is least confidence belong to level 4, for which there is support from only 1 source.

### Topology of the interaction network

We tested the properties of the network that is formed by the DNA-binding dimerising interactions, by plotting the log of frequency of proteins with K interactors against the log of K interactors (see Methods section and interactions_distribution worksheet in Additional file 1). We found that its distribution of connectivity decays in a similar fashion to a scale-free network (log-log plot linear regression R^2 ^= 0.654). This means that there are a few highly connected proteins-hubs (the RXRs [NR2B]) and many but poorly connected proteins that comprise the peripheral members of the network. A similar observation can be made for another dimerising network of TFs, the bHLHs [29]. The same kind of distribution was observed for the network that is formed by the non-DNA-binding dimerising interactions, where the hub was SHP [NR0B2] (see interactions_distribution worksheet in Additional file 1); the log-log plot linear regression R^2 ^= 0.7427. Nevertheless, this statistical property was not observed when the two networks of NR interactions were merged into one (log-log plot linear regression R^2 ^= 0.3935).

The overall topology of the NR protein-protein interaction network that we constructed is in good agreement with several reviews [5,32,3], a previous analysis that was performed manually [33] and the two analyses by the Koegl group. During the revision process of this manuscript, we also observed that the network is in good agreement with recent and extensive reviews on nuclear receptors (from the special issue of Pharmacological Reviews) and still contains the most extensive interaction dataset [34-41].

Our own text-mining effort, and integration of all data sources, confirms the overall hub-based structure and the central role of RXR [NR2B], which is the common heterodimerising partner of 11 phylogenetic groups. However, this new analysis also highlights the central role of SHP [NR0B2] as an additional hub, which suppresses the function of 10 NR phylogenetic groups when it interacts with them. In a sense, SHP [NR0B2] functions as a master negative switch due to the lack of a DBD and the presence of a repressor domain [42,43]. There is a distinct difference between the two hubs, RXR [NR2B] and SHP [NR0B2]. RXR interactions, mediated by the dimerisation helix 10(11) of the ligand-binding domain, are true NR dimerisations and are a pre-requisite for DNA-binding. In contrast, SHP [NR0B2] interactions with NRs do not involve the dimerisation domain but require short NR-binding domains (LXXLL, NR-box) within SHP and the AF-2 coactivator-binding surface within the NR ligand-binding domain. In a sense, SHP is a co-repressor hub. We compared this promiscuous interaction pattern of SHP with two well-known non-NR co-repressors, NCOR1 and SMRT, and found that these proteins also bind a large number of NRs (24 and 23 NRs respectively).

Our analysis reveals that the network is even more connected than was originally thought. Many interactions exist among peripheral members that are not covered in any generic publicly available database, like HPRD. This could be an effect of the distribution of information in the literature. Pubmed retrieves over 70,000 articles when querying with the keyword "nuclear receptor" and a certain sub-set of interactions are repeatedly mentioned, whereas a large number of interactions are mentioned very few times. This fact makes it extremely challenging for any researcher to detect rarely mentioned interactions, assemble a complete view of the interaction map, and update it with new data. To our knowledge, this paper presents the most thorough interaction dataset for NRs compiled to date and also identifies interactions of high confidence.

### Gene expression validates interaction data and shows a wide tissue expression pattern for NRs

As a quality control of this interaction dataset, gene expression data from normal human and mouse tissues were analysed. We verified that both partners of 99 out of 125 heterodimerising protein interactions with specific names in human and 87 out of 125 in mouse were present in at least one of the tissues (see Additional files 1 and 3). For most (15/26 in human; 36/38 in mouse) of the remaining heterodimerising interactions, one of the interacting partners was not present in any tissue. This may well be due to high-stringency criteria in our gene expression data analysis (see Methods). The above findings verify the biological significance of the NR interaction network.

The integration of gene expression, protein-protein interactions, and phylogenetic information also indicates the possibility that not all NR interactions have been verified yet. If there are at least two protein-protein interactions among any two phylogenetic groups, then other members of these groups could possibly interact if they are also co-expressed. This is the case for 119 pairs of NR proteins for which no interaction is reported. In human, there is at least one tissue where both interacting partners are present in the case of 82 out of 119 predicted interactions (Additional file 1). In mouse, this number rises to 85 out of 119 predicted interactions. Thus, we predict that a significant number of these 82 and 85 pairs of NRs, (with an overlap of 75 pairs between mouse and human) form biologically significant dimers, which remain to be validated.

Twenty human and 19 mouse NR genes are expressed on average in each of the 61 and 39 normal human and mouse tissues respectively. Moreover, 7 NR phylogenetic groups (NR1A, NR1B, NR1D, NR2B, NR2C, NR2F, NR3C) have at least one paralog (for every group) expressed in all 61 human tissues. This wide expression pattern is confirmed in 5 of these 7 families (except NR1D, NR2C) in mouse, where, there exists at least one paralog (for every group), expressed in more than 90% (36/39) of the tissues. The widely expressed NR genes and their highly connected network, where the hub RXR [NR2B] is always present, apparently create a competitive environment for the binding of peripheral members to the hub protein. This extremely competitive environment could be related to the fact that many NR dimers are ligand-activated, thus introducing an additional check-point in the network, in order to reduce noise.

### Protein-DNA interactions and negative feedback loops in the NR network

**Figure 2:**
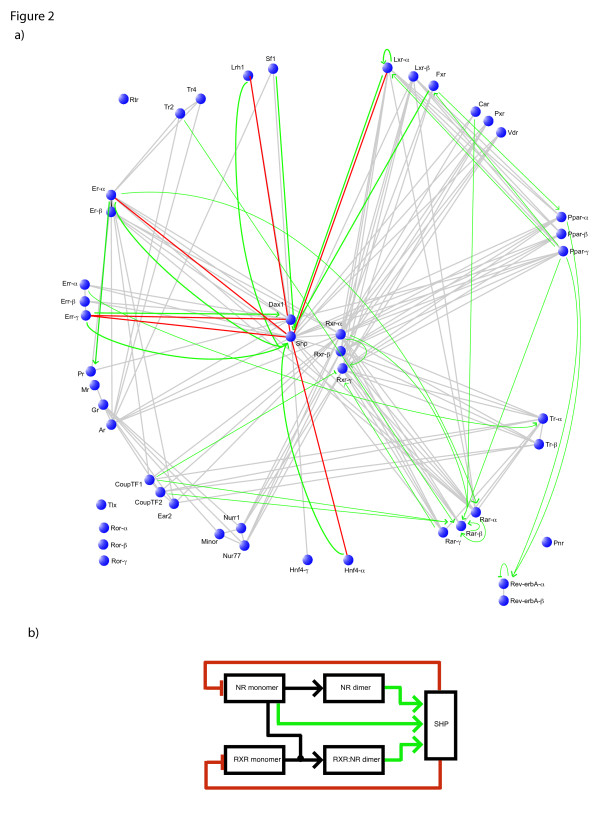
**Negative feedback loops in the NR network**. a) The protein dimerisation and protein-DNA interaction network of the NR family. Nodes represent proteins, grey edges represent protein-protein interactions and green edges represent protein-DNA interactions. Red edges represent protein-protein interactions that participate in the SHP negative feedback loops. b) The feedback loops are composed of protein dimerisation (black), protein-DNA interactions (activation: green), and inhibition through protein interaction (red).

Protein-DNA interactions among human NR genes were also retrieved (see Figure 2, Methods, and Additional file 1), in order to obtain a better understanding of the NR network. The integration of protein-protein and protein-DNA interactions reveals that there may be several negative feedback loops that all share the same component: the co-repressor hub, SHP [NR0B2]. The activation of several of the peripheral members (LRH1 [NR5A2], ER-a [NR3A1], ERR-g [NR3B3], HNF4-a [NR2A1], LXR-a [NR1H3]), will create functional dimers or monomers. These functional proteins in turn, will activate SHP. SHP will then suppress the activating effect of these peripheral members by binding to them (Figure 2). Due to the high sequence identity of the DBD, more than one member from each NR phylogenetic group (SF1 [NR5A1], ER-b [NR3A2], ERR-a [NR3B1], ERR-b [NR3B2], HNF4-g [NR2A2], FXR [NR1H4], LXR-b [NR1H2]) could regulate the promoter of SHP. This, combined with a conserved interaction domain for SHP, could increase the number of negative feedback loops from 5 (observed) to 12 (predicted by sequence identity). The possibility of SHP participating in negative feedback loops has been raised previously, but not to such an extent [44]. Negative feedback loops are essential for establishing oscillations in transcription [45]. It has been reported that such loops occur at the post-transcriptional level, where the protein-DNA interactions are thought of as the "slow" part (often having a timescale of minutes), whereas the protein-protein interactions can be thought of as the "fast" part (often with a subsecond timescale) [46]. It is intriguing that this negative feedback loop pattern was not observed for the dimerisation hub RXR [NR2B]. It appears that the negative feedback loops occur at the co-repressor level and not at the dimerisation level. Regulation at the co-factor level has previously been highlighted as a very important factor of gene regulation [47].

## Discussion

The genomic era has revealed that dimerising TFs are at the "heart" of animal complexity and deserve a lot of attention, in order to deconstruct and understand the major control circuits of life. It has been observed that the increased complexity of organisms correlates with an increase in control functions per gene; i.e. both the absolute number of TFs and the ratio of that number to the number of genes controlled are increased [48-51] as organisms become more complex. Moreover, hetero-dimerisation has proved to be an efficient and successful strategy for increasing the complexity and range of gene regulation, since it combines already available factors in new control programmes [52,45]. A better understanding of regulatory networks has been achieved in the last few years by studying their architecture, motifs, function and evolution (either by gene duplication or by accruing regions of disorder) [46,53-55].

Previous work on the largest eukaryotic family of dimerising TFs (over 100 genes in humans), the bHLH family, has revealed that they form complex and conserved dimerisation networks that are not random, but hub-based [29]. Such topologies evolved by single-gene duplications, domain re-arrangements and possibly whole-genome duplications [29] (i.e. the two rounds of whole genome duplication – 2R hypothesis – at the origin of vertebrates [56]). We observed that the topology of the dimerisation network for the second largest eukaryotic family of dimerising TFs (over 50 genes in humans), the bZIPs, is also not random, although it is not hub-based. The overall architecture is linked to redox control of DNA binding [57]. Again, this family has a similar evolutionary history [58]. In the current study, we focused on the third largest family of dimerising TFs, the NRs, and we observed that they have a hub-based topology, like the bHLHs. For the interactions that form DNA-binding dimers, RXR [NR2B] is the common heterodimerising partner. For the interactions that form non-DNA-binding dimers, SHP [NR0B2] is the common partner, and functions as a co-repressor. It is intriguing that when, the two networks were studied separately, they revealed their scale-free nature. Nevertheless, when they were merged, this property was lost (see Methods section and interactions_distribution worksheet in Additional file 1).

Since the bHLH and NRs are dimerising TFs that also share a hub-based topology, it is interesting to compare the two families and their networks in order to reveal common features, which could be universal, as well as unique features that characterise each network.

### Similarities between the bHLH and NR dimerisation networks

Fast information flow as a result of high connectivity and hub-based topology [19] seem to be key elements of the NR and bHLH [29] dimerising networks. Furthermore, these two networks have both evolved proteins that lack the DBD (the Id and SHP [NR0B2] proteins for the bHLH and NR networks, respectively). Id and SHP both retain the dimerisation domain and thus inhibit DNA binding when they interact with the hub protein (preferably), or even with a protein that is a peripheral member of the network. These two networks share not only their topological features but also their evolutionary histories, since they both emerged by two waves of gene duplications – at the origin of metazoa, and the origin of vertebrates. Step-wise gene duplications, that lead to new binding specificities and regulation, are a common mechanism in protein complexes [59]. Alternative models of network evolution also exist, e.g by increasing the protein length, or acquiring regions of disorder and/or internal repeats [55]. It appears that evolution favoured the hub-based topology in both cases.

### Differences between the bHLH and NR dimerisation networks

Despite these similarities, the two dimerising networks have a number of distinct features. Although RXR [NR2B] is a potent dimerisation hub, NRs have a great ability to bind DNA as monomers or homodimers. In addition, we observed a large number of interactions between peripheral members that were verified both by text-mining and Y2H approaches. In the bHLH network, on the other hand, heterodimerisation with the hub is the essential control mechanism. Therefore, the role of the hub seems more important in the bHLH network. This results in the network being vulnerable to mutations in the gene encoding the hub protein. Nevertheless, the role of the peripheral members should not be underestimated: when they dimerise with the hub, they regulate a large number of genes, thus becoming hubs as well in the overall genetic network. Furthermore, the efficiency of the bHLH network is increased since it allows suppression of a large number of peripheral members by suppressing only one protein, the hub.

In the NR network, the ability of several peripheral members to be functional either as monomers or homodimers makes them less dependent on the hub. This may explain why two major mechanisms of suppression have evolved. One uses the COUPTF [NR2F] repressor proteins, that compete with several RXR [NR2B] heterodimers for common DNA-binding sites [60]. The second exploits the emergence of an NR member (SHP [NR0B2]) that lacks DNA-binding ability, contains inhibitory domains, and not only interacts with the hub, but also interacts with peripheral members that are not entirely dependent on the hub. It would be reasonable to assume that the second mechanism of repression would be the prevalent one. It has been reported, based on RT-PCR experiments [61], that SHP [NR0B2] is ubiquitously expressed in rat tissues. Paradoxically, however, from our human gene expression data, it appears that COUPTF [NR2F] is more widely used than SHP. Possibly, the highly stringent parameters that we used in our gene-expression analyses underestimated the tissue distribution of SHP [NR0B2]. Nevertheless, the wide gene expression profile of COUPTF [NR2F] and the restricted gene expression profile of SHP are additionally supported by Q-PCR experiments in mouse tissues [2].

One of the advantages of a hub-based topology is economical control at the genome level, since inhibition of the whole system can be easily achieved by evolving a minimal number of repressors that target the hubs.

Data integration and static modelling are only the first steps in this new era of "omics" and systems biology. They allow us to capture a snapshot of the global picture, understand the properties of the system as a whole, generate new hypotheses (like prediction of protein-protein interactions) and perform, in the future, targeted experiments. Quantitative measurements of protein-protein, protein-DNA binding affinities, and mathematical modelling should be the next steps that would allow us to comprehend, to an unprecedented extent, the biology of nuclear receptors.

## Conclusion

We infer that the topology of this network is hub-based, and much more connected than previously thought. The hub-based topology of the network and the wide tissue expression pattern of NRs create a highly competitive environment for the common heterodimerising partners. Furthermore, a significant number of negative feedback loops is present, with the hub protein SHP [NR0B2] playing a major role. We also compare the evolution, topology and properties of the nuclear receptor network with the hub-based dimerisation network of the bHLH transcription factors in order to identify both unique themes and ubiquitous properties in gene regulation. In terms of methodology, we conclude that such a comprehensive picture can only be assembled by semi-automated text-mining, manual curation and integration of data from various sources.

## Methods

### External protein interaction databases

The most comprehensive and publicly available protein-protein interaction database, HPRD, [18,31] was scanned for dimerising interactions among NR proteins.

### Extraction of protein-protein and protein-DNA interactions from the literature

For the extraction of protein-protein interactions from the literature (comprising both abstracts and full text), the following methodology was used:

1) We used an established classification scheme for the human NRs (proposed by nuclear receptors Committee, 1999) and based on sequence identity (see Additional file 1).

2) Synonyms for every NR protein were retrieved from GeneCatalogue, an AstraZeneca gene and protein reference database, and from a review paper [32] (see synonyms worksheet in Additional file 1).

3) The QUOSA software was used to retrieve full-text articles and abstracts from MEDLINE that referred to NRs [62].

4) A keyword term ("nuclear receptor") was identified that would retrieve the highest number of relevant articles with the QUOSA software.

5) The following were downloaded:

a. The relevant 5,241 full text articles of 2003 in PDF or HTML form, depending on availability.

b. The relevant 46,300 abstracts of the 13 years from 1993 to December 2005 in plain text form.

6) Full-text documents were converted from PDF and HTML into plain text format, using the freely available MULTIVALENT [63] and HTMLESS software [64].

7) PERL scripts were written to extract sentences where two different NR protein names or any of their synonyms co-occurred with terms that described an interaction (*e.g. *"dimer", "interact", etc).

8) All (~3500) sentences retrieved from full text and (~3000) sentences retrieved from abstracts were read manually and those that described a physical or protein-DNA interaction were marked (see Additional files 1 and 2).

9) While reading the extracted sentences, we earmarked dimers that were shown to bind to DNA. In addition, the Nuclear Receptors Factsbook was scanned to identify dimers that bound to DNA. If a dimer between two phylogenetic groups was shown to bind to DNA, any other dimer among proteins of the same two phylogenetic groups was predicted to bind to DNA as well, due to the very high sequence conservation of the DNA-binding domain. For example, a DNA-binding dimer is formed between RXR_b [NR2B2] and RAR_b [NR1B2]. Based on this fact, we predict that the dimer formed between RXR_g [NR2B3] and RAR_b [NR1B2] also binds to DNA.

10) A large number of sentences from the full-text subset did not provide the specific names of the interactors (due to PDF conversion) and therefore were not used subsequently for the dimerisation dataset.

11) From the 46,300 abstracts, 1128 abstracts contained 1802 sentences that mentioned a true protein-protein interaction.

12) Graphs of protein-protein and protein-DNA interactions were generated using the Adobe Illustrator software. Matrices of protein-protein interactions and co-expression were generated using a customized version of the R gplots package [65].

### Integration of protein interaction data from different species

Protein interactions among mammalian members of the NR family were identified. Protein interactions for mammals were extracted for both human and murine orthologues, due to their high amino-acid sequence identity (>70%). Identification of murine-human orthologues was based on literature reports. Interactions from different species were collapsed on the same graph as in [29]. For example, the human A-factor_(human) _- B-factor_(human) _and mouse B-factor_(mouse) _- C-factor_(mouse) _interactions were collapsed into the mammalian A-factor_(mammalian) _- B-factor_(mammalian) _- C-factor_(mammalian) _interactions.

The validity of this approach has been verified by cases where interactions are conserved even when one of the heterodimeric partners is the ortholog from another distant species [66].

### Statistical properties of the protein-protein interaction network

In order to assess whether a network is scale-free or not, the distribution of connectivity is plotted (see interactions_distribution worksheet in Additional file 1). Specifically, we plotted the log of the frequency of proteins with K interactions, versus the log of K interactions. A network may resemble a scale-free topology if the distribution of connectivity decays in a power-law fashion. Therefore, the better the trendline (in the log-log plot) fits a linear regression, the more the network resembles a scale-free topology. For the DNA-binding and non-DNA-binding dimers, we obtained an R^2 ^value of 0.654 and 0.7427 respectively, concluding that the networks resemble a scale-free topology. For the whole network (adding the DNA and non-DNA binding dimers together), we obtained an R^2 ^value of 0.3935, concluding that the network does not resemble a scale-free topology.

### Protein-DNA interactions

While mining the full-text literature from 2003, any document that mentioned protein-DNA interactions between any two NR members was also marked (see Additional File 1). In this paper, the term "protein-DNA interaction" is meant to denote the binding of one TF to the upstream regulatory DNA element of another NR gene; that could either activate or repress its expression. The whole articles were read, and their references followed, in order to verify the 19 protein-DNA interactions. We also expanded this incomplete dataset to a total of 28 protein-DNA interactions by looking in the nuclear receptor Factsbook [67].

### Gene expression data

In order to better understand the mechanisms employed by the NR family, gene expression data were used i) for the 48 human NR genes (provided by AstraZeneca) and ii) for the 49 mouse NR genes, by mining the published dataset of Bookout *et al *[2].

The human gene expression dataset was based on the Affymetrix HG_U133 chip. The focus was on expression of probe sets that mapped to an exon or a 3'UTR in tissue samples that were classified as having normal morphology and pathology over 99 different human tissues (see Additional file 3). The Affymetrix MAS5 algorithm uses the Detection Call as a qualitative measure of gene expression. In any single experiment, a probe set may be called Present, Marginal or Absent. The presence or absence of a gene's transcript from a tissue in this analysis was determined according to the following rule: if the tissue had more than 10 experimental samples and the gene transcript was called Present in at least 50% of the samples, the transcript was considered present. If there were less than 10 experimental samples for a tissue, the presence or absence of the transcript of any gene for that particular tissue was considered undetermined. After applying the above criteria, we found 42 out of 48 human NRs present in at least one of 61 normal human tissues.

The mouse gene expression dataset was based on Q-PCR experiments, performed by Bookout et al., [2], for all 49 mouse NR genes, over 39 normal tissues, repeated in two mouse strains (C57Bl/6J & 129x1/SvJ). The presence of a gene's transcript from a tissue in this analysis was determined according to the following rule: the relative mRNA level of a given transcript had to be above the suggested cut-off of 0.1, in both strains. After applying the above criterion, we found transcripts of 41 mouse NR genes present in at least one of the 39 normal mouse tissues.

## Authors' contributions

GDA participated in the design of the study, carried out the text-mining, participated in the manual curation, integration of data from all sources and helped to draft the manuscript. EEP participated in the design and coordination of the study, in the integration of data from all sources and helped to draft the manuscript. NM carried out the analysis of human gene expression data. AI participated in the manual curation of protein-protein interaction data. MRR, EBB, DLR participated in the design of the study and helped to draft the manuscript. DDG participated in the conception and design of the study. SGO conceived of the study, and participated in its design and coordination and helped draft and revise the manuscript. All authors read and approved the final manuscript.

## Supplementary Material

Additional file 1 NR Interactions. It contains in 6 worksheets i)the Nomenclature and synonyms of NRs, ii) the protein-protein interactions, their source, the level of confidence, in how many tissues they are co-expressed, iii) the same as previous worksheet, but each interaction mentioned only once, not in both directions, iv) the predicted protein-protein interactions and in how many tissues they are expressed, v) the analysis of distribution of connectivity for the NR protein interaction network, vi) the protein-DNA interactions. Click here for file

Additional file 3 Tissue presence of NRs. It contains in 4 worksheets, i) the presence or absence of NRs in the 61 normal human tissues, ii) the number of human tissues where any potential pair of NRs is co-expressed, iii) the presence or absence of NRs in the 39 normal mouse tissues, iv) the number of mouse tissues where any potential pair of NRs is co-expressed. Click here for file

Additional file 2 Sentences from text-mining. It contains all the manually curated sentences that describe a protein-protein interaction, and the PUMED identifier of the relevant document. Click here for file
